# 

**Published:** 1888-08-18

**Authors:** 


### "THE HOSPITAL" LIBRARY OF NEW AND STANDARD WORKS.

For all information respecting the appearance of Titles, Prices, etc., of Books in this page, apply to Mr. W. H. HOWE, office of THE HOSPITAL, 38, Old Jewry.

11th edition, 2s, post free for 26 stamps.

STAMMERING, STUTTERING, LISPING, &c.; their Causes and Cure. By W. ABBOTTS, M D., Physician to the Hospital for Speech Affections.

BEAUMONT and Co., 6, Wells Street, Oxford Street, W. (72)

Crown 8vo., cloth, 2s 6d.

THE COMMONER DISEASES AND ACCIDENTS TO LIFE AND LIMB. Firstly, their Prevention; secondly, their Immediate Treatment. By M. M. BASIL, M.A., M.B., C.M. (Edin.).

"Brief, clear, and good."—*Athenæum.*

"Thoroughly readable."—*Hospital.*

London: J. and A. CHURCHILL, 11, New Burlington Street.

### Medical Library.

ABDOMEN; Diseases of the. By S. O. Habershon. 21/- *Churchill.*

CANCER: Its Allies, and other Tumours. By F. A. Purcell 10/6 *Churchill.*

DIPHTHERIA: Its Nature and Treatment. By Sir Morell Mackenzie. 5/- *Churchill.*

EAR: The Diseases of the. By A. Hartmann. 9/- *Pentland.*

EYE: On Diseases of the. By C. B. Taylor. *Redway.*

HEART: A Practical Treatise on Diseases of the. By W. H. Walshe. 16/- *Smith and Elder.*

HELPS to Health. By Hy. C. Burdett. 1/6 *Kegan Paul.*

HELP in Sickness. By Hy. C. Burdett. 1/6 *Kegan Paul.*

LIVER: Diseases of the. By Geo. Harley. 21/- *Churchill.*

LUNGS: A Practical Treatise on Diseases of the. By W H. Walshe. 16/- *Smith and Elder.*

MIDWIFERY FOR MIDWIVES. A Manual of. By Fancourt Barnes. 6/- *Smith and Elder.*

NERVOUS SYSTEM; A Manual of Diseases of the. By W. R. Gowers. 12/6 *Churchill.*

### Library of Biography and History.

BACON. By R. W. Church (Dean of St. Paul's). 5/- *Macmillan.*

GLADSTONE; Anecdotes of. By an Oxford Man. 1/- *Hamilton.*

GREECE: A History of. Part I. By Edwin Abbott. 10/6. *Rivingtons.*

HENRY II. By Mrs J. R. Green. 2/6. *Macmillan.*

MITCHELL: Life of John. By Wm. Dillon. 2 vols. 21/- *Kegan Paul.*

### Library of Christian Literature.

A CENTURY OF CHRISTIAN PROGRESS AND ITS LESSONS. By J. Johnston. 3/- *Nisbet*

FAITHFUL DEPARTED: The Present State of the. By Rev. Geo. Body. 1/- *Masters.*

ST. JOHN, THE AUTHOR OF THE FOURTH GOSPEL. By H. H. Evans. 6/- *Nisbet.*

THE VICTORY OF THE CROSS. By B. F. Westcott. 3/6 *Macmillan.*

### Dictionaries and Encyclopædias.

EVERYBODY'S POCKET CYCLOPÆDIA. By E. Sheldon. 6d. *Saxon and Co.*

MIDDLE ENGLISH: A Concise Dictionary of. By A. L. Mayhew—W. W. Skeat. 7/6 *Froude.*

NATIONAL BIOGRAPHY: Dictionary of. By Leslie Stephens 15/- *Smith and Elder.*

NEW ENGLISH DICTIONARY ON HISTORICAL PRINCIPLES. Edited by James Murray. Part 4 (Bra-Cass). 12/6 *Froude.*

### Library of Fiction.

A BITTER REPENTANCE. By Lady Virginia Sandars. 3 vols. *Hurst and Blackett.*

A WOMAN'S FACE. By F. Warden. 3 vols. *Ward and Downey.*

CHILDREN OF GIBEON. By Walter Besant. 2/- *Chatto and Windus.*

EVE. A Romance. By Author of "John Herring." 2 vols. *Chatto and Windus.*

JESS. By H. Rider Haggard 2/6 *Smith and Elder.*

### To Secretaries and Matrons.

THE Editor will be glad to have the Regulations and each Report as issued by Asylums, Hospitals, Convalescent and Nursing Institutions, as well as those of other Charities, and an early copy in duplicate of all Appeals and Festival Notices, etc., addressed to *The Editor, The Lodge, Porchester Square, London, W.* Any superintendent, secretary, matron, or other chief official who regularly sends such information may be registered, on application to the Publisher, to receive free of cost a cover for binding THE HOSPITAL in half-yearly volumes.

### Notice to Advertisers and Subscribers.

All Advertisements and Business Communications should be addressed to The Publisher, *not to the Editor*, THE HOSPITAL, 38, Old Jewry, E.C. Terms for Advertising given on page iii.

### The Student's Column.

### UNIVERSITY OF EDINBURGH.

The following is the official list of candidates who passed the first professional examination in July :—

Walter Adam, R. E. Adamson, E. H. Alexander, W. G. Alexander, G. B. Anderson, James Anderson (M.A.), W. H. Annan, R. H. Armstrong, I. C. Atkinson, L. J. Barbeau, H. P. Barlow, W. F. Bauchop, Hight Blundell, J. J. Brennan, T. B. Brierley, George Butters, A. N. S. Carmichael William Carr, C. W. Chapman, P. G. Cilliers, G. A. Cohen, James O. Coker, J. W. Compton, John Cowan, J. N. Craig, Richard Davies, J. W. Dawson E. C. Dobie, C. W. Donald, R. H. Drennan, Alfred Duke (M.A.), J. A. H. Duncan, C. C. Easterbrook (M.A.) (with distinction), Samuel Edgerley (M.A.), C. R. Edmondson, R. E. Evans, R. S. Ferguson, R. W. L. Fernandez, J. L. Ferris, S. B. Figgis, L. G. Fink, H. W. Fisher, E. G. Fortune, J. S. Fowler, W. T. Fox, M. H. Foye, H. E. Fraser (M.A.), E. B. Fuller (with distinction), R. B. Gass, S. A. Gibbs, C. J. Gomes, P. C. Govinden, W. H. Gregory, Frank Grenier, Harry Grey, G. K. Grimmer, W. C. Grosvenor, A. A. S. Harriss, S. H. Hartley, Edmund Hay, H. H. Hearsey, Alexander Hendry, Sidney Hillier, A. J. Hughes, G. F. Hulme, H. S. W. Jones (with distinction), H. B. Jones, J. A. Kennedy, Edward Kinmont, H. B. Knox, J. E. Knox, L. J. Lamrock, G. R. Leighton, R. M. Leslie (with distinction), J. R. Liddell, E. S. Littlejohn, John M'Clymont, A. G. Macdonald, A. M. M'Donald, T. N. Macgowan, D. J. Mackenzie, W. C. Macknight, James Maclean (M.A.), Samuel Maclean, David Macmillan (M.A.), G. W. F. Macnaughton, D. A. C. Mac-Phail, H. B. Mapleton (B.A.), Charles Martin, T. G. Matthews, A. J. Meikle, David Melville, R. T. Mitchell, James Miller, W. C. Milroy (M.A.), G. H. Mitchell, L. P. More, P. St. C. More, D. S. Morrison, J. D. R. Munro, William Murray, Alexander Van Niekerk, Samuel Oddie, A. A. O'Hara, T. L. Parry, C. H. Passman, D. W. H. Paterson, T. W. Pattinson, R. O. Petrie (M.A.), John Phyn, W. H. Pimblett, F. L. Pochin, W. G. Putnam, M. M. Rattray, G. P. Richards, David Ritchie, J. J. Roberts, H. A. Robertson, J. F. Robertson, William Rogers, David Rorie, A. F. Rosa, A. S. Rose, Hugh Ross, M. J. L. E. Rouillard, J. G. V. Sapp, J. B. Scott, P. W. Shaw, S. A. Shiach, J. W. Shiels, A. T. Simpson, A. S. Smith, C. E. Stephens, Riccardo Stephens, W. J. J. Stewart, W. S. Syme, J. M. Tait, George Templeton, G. D. S. Thom, Bernard Thomas, A. C. Thomson, J. T. Thyne, Walter Tibbits, F. R. Van Langenberg, H. W. Vaughan, Williams, Andrew Walker, H. S. Walker, T. D. Walker, W. J. Walker, G. S. Walton, Godfrey Warnes, R. H. Watson, Thomas Watson, W. T. Wearing, E. J. Weightman, A. J. Wheatley, J. H. G. Whiteford, Harry Whittome, David Weild (M.A.), J. D. Williams, J. W. Williams, R. M. Williams, A. G. Wills, J. T. Wilson, R. H. Wilson, T. A. M. Wilson, W. H. Wilson, J. B. Yeoman.

### THE DEGREES OF BACHELOR OF MEDICINE AND MASTER IN SURGERY.

*Small Capitals indicate that the Candidate has passed the Examinations with First-Class Honours.*

*Italics indicate that the Candidate has passed the Examination with Second-Class Honours.*

Robert Abernethy, Scotland, James Alexander Adamson, Scotland, Robert Andrew, Scotland. Edward Farr Armour (M.A.), Scotland. Richard Arthur (M.A.), Scotland. Alexander Asher, Scotland. Louis Edward Barnett, New Zealand. John Hargreaves Battersby, England. Jas. Arnold Burger Bayly, Cape Colony. Lodewijk Andries Willem Beck, Cape Colony. Henry Anthon Becker, Orange Free State (received the degrees on 26th November, 1887). *William Andrew Betts*, England. Arthur James Spowart Beveridge, England. James Roderick Bird (B.A.) Canada. Richard Dalby Booth, England. Richard Laing Booth, India. John Thomas Borthwick, Australia ; Norman Laurence Boxill (B.A.), Barbados ; Francis Darby Boyd, Scotland ; James Whiteside Bridges (B.A.), Canada ; Reginald Broadbent, England ; George M'Murdo Brown, Scotland ; James Kemp Brown, England ; Arthur William Treminheere Buist-Sparks, Scotland ; Arthur Edwin Bullock, England ; Alfred Charles Burnell, Calcutta ; Thomas Wm. Butcher, England (received the degrees on 18th April 1888) ; George Clark Cameron, Scotland ; Thomas Vincent Campbell, (M.A.), Ireland ; Frederick Harold Carlyon, England ; David Bell Carse, Scotland ; William James Cattan (B.A.), New Zealand ; Charles Henry Temple Chevallier, England ; John Cockton, England ; Michael Waistell Wilmshurst Cowan, England ; James Muir Crawford, Scotland ; Edward Carter Cridland, England ; Arthur John Wilson Dalzell, England ; John Henry Deamer, England ; Russell John Drummond, India ; Charles Wm. Duggan, Scotland (received the degress on 26th November 1887) ; Henry Arnold Eaton, England ; Thomas Watts Eden, England ; *Robert Edie*, Scotland ; Frank Ashby Elkins, England ; Andrew Elliot (M.A.), Scotland ; William Ernest Lloyd Elliott, Wales ; Richard Cogswell Elsworth, England ; John Alfred Ewan (M.A.), Scotland ; Charles Samuel Facey, England ; Charles Christie Fleming, Scotland ; Robert Alexander Fleming (M.A.), Scotland ; Charles Melville Flide, Australia ; William Fordyce (M.A.), Scotland ; James Alexander Forrest, Australia ; Frederick Charles Julius Fulss, Cape of Good Hope ; John Hill Ross Garson, Scotland ; Richard Julyan George, England ; Alexander Lockhart Gillespie, Scotland ; Andrew Gray (M.A.), Scotland ; Curt Grobbelaar (B.A.), South Africa ; Alexander Chorley Hall, England ; Arthur Conning Hartley, Scotland ; Charles Edward Harvey, Jamaica ; Thomas Daniel Hill Holmes, England (received the degrees on 26th November, 1887) ; Walter Hume, Scotland ; Timothy Augustine Hynes, South Australia ; James Conway Jameson, Scotland ; *Sydney Jamieson* (B.A.), Australia ; Charles Arthur Johnston, India ; David John Jones, Wales ; William Watkins Jones, Wales ; George Kelman, Scotland ; Patrick James Kenna (B.A.), Australia ; Thomas Looney Kennish, England ; Eustace Julian Keogh, Australia ; Alexander Livingstone Kerr, Australia ; William Kinnear (M.A.), Scotland ; Olaf Kloster, Norway ; George Knowles, England ; John Elisha Kuhne, Switzerland ; Daniel Johannes Kuys (B.A.), Cape of Good Hope ; Henry Christopher Lamport, Natal ; James Millar Laughton, Scotland ; *Stephen Moister Laurence*, West Indies ; Samuel Frank Lautré (B.A.), South Africa ; Arthur Septimus Lawrence, South Africa ; *William George Laws*, England ; John Liddell (M.A.), Scotland ; William Lockwood, England ; James Alfred Lowson, Scotland ; William Lundie (M.A., B.Sc.), Scotland ; *Frederick John M'Cann*, Scotland ; Alexander Macdonald, Scotland ; James M'Donald, Wales ; Alexander James MacGregor, Scotland ; George Scott MacGregor, Scotland ; Thomas MacGregor, Scotland ; John Robertson M'Intosh, (B.A.), Canada ; Æneas Donald Mackay Macintyre, Scotland ; Alexander Maclean Mackay, Scotland ; David James Mackay, Scotland ; Donald MacLeod, Scotland (received the degrees on 18th April, 1888) ; William Grant Macpherson, Scotland ; Christopher Martin, England (received the degrees on 26th November, 1887) ; Kenneth Maxwell, Tasmania ; Thomas Christopher Meikle (M.A.), Scotland ; *James Middlemass* (M.A., B.Sc.), Scotland ; Alexander Miles, Scotland ; George Victor Miller, England ; Vincent Milner, England ; John Gay Moffat, Scotland ; John Montgomery, Scotland ; Frederick William Gisborne Morgan, Ceylon ; Thomas Howard Morgan, Wales ; Reginald Herbert Morrison, Australia ; Charles Frederick Arrowsmith Moss, England ; Robert Muir (M.A.), Scotland ; Neil Gordon Munro, Scotland ; Patrick Murison, Scotland ; Charles David Musgrove, England ; John van Niekerk, Cape Colony ; John Nightingale, England ; Augustus Beachamp Northcote, England ; Charles Henry Leet Palk, England (received the Degrees on 26th November, 1887) ; John Charles Palmer, Australia ; Thomas Clerkson Paterson, Scotland ; Benjamin Lewis Paton (B.A.), India ; Julius Petersen, South Africa ; John Phillips, England ; Kanta Prasad, India (received the degrees on 26th November, 1887) ; John Trevor Pritchard, England (*in absentia*) ; John Randle Sierra Leone ; Joseph Riley Ratcliffe, England ; James Carson Rattray, Scotland ; Edward William Rayment, England ; Arthur Gordon Reid (B.Sc.) Canada ; Charles Reid, Scotland ; Robert Renton, Scotland ; William James Richardson, Scotland ; Richard Richmond, England ; *James Ritchie* (M.A.), Scotland ; Andrew Robertson (M.A.), England ; Charles Robertson, India ; Robert Robertson, Scotland ; John William Rodgers, England ; Donald Mars Morphett Ross, Scotland ; Robert Russell Ross, Nova Scotia ; James Russell (M.A.) Scotland ; Robert Arthur St. Leger, Cape Colony ; Gerald Septimus Samuelson, England ; Matthew Brown Saunders, England ; Charles Butler Beck Savory, England ; James Foster Scott (B.A.), U.S., America ; William Affleck Scott (M.A.), Scotland ; Robert Brownlee Shaw, Scotland ; George Shields, Scotland ; John Howard Slayter, Canada ; Charles Allen Casterton Smelt, England ; Edmund Moody Smith, England ; James Smith, Scotland ; John Howie Smith, Scotland ; *James Smuts*, Cape Colony ; William Smyth, Ireland ; *John Somerville*, New Zealand ; Gabriel Hendrik Steyn, Cape Colony (received the degrees on 26th November, 1887) ; Hendrik Vos Steyn, Cape Colony (received the degrees on 26th November, 1887) ; William Dunbar Sutherland, India ; George Temple Tate, England ; George Henry Temple, England ; Carel Theodorus Te Water, South Africa ; Ieuan George Thomas, Wales ; *Isaac Thompson*, England ; Arthur Thomson, Canada ; Albert Edward Thomson (B.A.), Canada ; *John Christopher Thomson* (M.A.), Scotland ; William Tweeddale, Thomson, Scotland ; John Townsley, Scotland ; David Traill (M.A., B.Sc.), Scotland ; *James Henry Traquair*, Scotland (*in absentia*) ; *Ninian George Trotter*, New Zealand ; *Dawson Fyers Duckworth Turner* (B.A.), England ; Herbert Vine, England ; Frederick Watson, Scotland ; William Muir Crawford Watson, Scotland ; John William Watterson, England ; *John Clarence Webster* (B.A.), Canada ; William Weir (B.Sc.), Scotland ; Ernest Nevile Keys Wells, England ; Andrew Westwood, Scotland ; Tom Brown White, Scotland ; Henry Townshend, Wickham, England ; *John David Williams*, Wales ; Alfred Maxwell Williamson, Scotland ; John Hay Wilson, Brazil (received the degrees on November 26th, 1887) ; Robert Appleton Wilson, England ; Robert Wise Scotland ; David James Wood, Scotland ; Henry Stotesbury Wood, India ; Ernest Adolphus Woodward, New South Wales ; Walter Wynne, Wales ; Robert Edward Burnett Yelf, England ; Thomas MacCubbin Young, Scotland.

The Ettles Scholarship has beed awarded to Thomas Watts Eden, M.B., C.M.

The Stark Scholarship in Clinical Medicine has been awarded to James Brown Bird.

The Beaney Prize has been awarded to John Clarence Webster, B.A., M.B., C.M.

The Buchanan Scholarship has been awarded to William Fordyce, M.A., M.B. C.M.

The James Scott Scholarship has been awarded to Thomas Watts Eden, M.B., C.M.

The Freeland Barbour Fellowship has been awarded to John David Williams, M.B., C.M.

The Gunning Victoria Jubilee Bell Prize in Physiology has been awarded to George Neil Stewart, M.A., D.Sc.

The Gunning Victoria Jubilee Lister Prize in Surgery has been awarded to Ernest Frederic Neve, M.D.

The Wightman Prize has been awarded to Arthur John Whiting.

### AMUSEMENTS WITH PROFIT FOR ALL AGES.

**Rules.**—All answers must be addressed to the PRIZE EDITOR, THE HOSPITAL, 38, Old Jewry, Cheapside, London, E.C., not later than the *first* post on Thursday next. The full name and address must in all cases be given, but a *nom de plume* may be added for publication. Only one side of the paper must be written on.

### FOR MEN AND WOMEN.

**Rule.**—Competitors can enter for *all* quarterly competitions, but no competitor may take more than *two quarterly prizes* during the year.

### First Quarterly Poetical Competition.

Commenced May 26th, 1888; ends August 25th, 1888.

This quarter a PRIZE of ONE GUINEA will be given to the competitor who gains the highest number of marks for original poems, on any subjects suitable for insertion in this paper. All contributions sent in will be credited according to merit, eighteen marks being the highest score for any one contribution. This competition to alternate weekly with the set acrostic competition.

### Acrostic Competition.

This quarter the original acrostic competition will be discontinued, and A PRIZE of HALF-A-GUINEA will be given to the competitor who gains the highest score for solution of acrostics set. *One mark* only will be given to the competitors who solve all the lights of each acrostic.

Exception will be made in the case of *quotation acrostics*, when *two marks* will be given, *one* for the correct solution of all *lights*, and *one* mark for the *source* of the quotations.

This week credit will be given for

### No. 6.—Double Acrostic.

You want an Acrostic? Then here's a prognostic,
Growl'd an old bachelor wrapt in the grumps:
What with groaning and wheezing, and coughing and sneezing,
My primals and finals are ditto for "dumps."

My voice so cunningly I frame,
Men go no wiser than they came.

Thus, while spellbound the boys remain,
Jack fights his battles o'er again.

Dearly I love thee, cause of all my woe;
I would deny thee, but I love thee so.

Leave coast and vale if him you seek;
Go search the hillsides of the Peak—
"A singular bird, with a manner absurd."

Thou art so near, O Lamp of Night,
Whene'er thou shinest in my light.

'Twas here the man of destiny was found,
A despot yesterday, to-day discrown'd.

Donna Duño What her name is?
"Saw ye ever such a maid,
With her feathers swaling o'er her,
"And her spangled rich brocade?"

With this phylactery of occult virtue
Nor ague nor disease shall hurt you.

### Answers.

### No. 5.—Double Acrostic.

S icknes S
I oni C
C hancer Y
K il T
L eec H
E conomiz E

Correct answers have been received from Dodo, Lady Clara, Alicia, Alice, Tinie, Ruffles, Mater, Reldas.

### Results of Third Quarterly Word Competition.

| Names. | Total of Marks. | Names. | Total of Marks. |
|---|---|---|---|
| Patience | 1,181 | Oliver | 470 |
| Tinie | 1,180 | Carmen | 396 |
| Lightowlers | 1,115 | Brisbane | 158 |
| Lauriston | 1 110 | Geranium | 124 |
| Sym | 1,055 | Ione | 107 |
| Reldas | 1 054 | Robe | 101 |
| Dodo | 1 046 | Pec | 31 |
| Puff | 953 | Dunce | 29 |
| Dollie | 717 | M. R. | 25 |
| Alicia | 487 | Edge | 20 |

We award First Prize of One Guinea to Patience, Miss C. F. Calise, Craven Road, Newbury. Second Prize 15s. to Tinie, Miss H. Todd. Craven Villas, Newbury Third Prize, 10s. 6d. to Lightowlers, Miss C Chadwick, 98, Huskisson Street, Liverpool.

### THE CHILDREN'S CORNER.

PRIZES.
FOR MOST CORRECT ANSWERS TO PUZZLES.
This Commenced October 1st, 1887.
One Pound a Month.
A PRIZE OF THREE GUINEAS
to the Competitor who shall have sent in the largest number of correct or best answers during the twelve months.

### Puzzles, Third Week.

No. 9. LETTER PUZZLE.—The following letters correctly placed will give the names of four celebrated statesmen:
a b e e f i k l l o o p p r t t u w x

No. 10. DOUBLE ACROSTIC.—Initials and finals form the names of a well-known Swiss mountain and town. 1. The first teetotaller. 2. Stored in winter for summer's use 3. A celebrated Welshman. 4. A distinguished German naturalist and physician. 5. A city and territory of Hindoostan.

No. 11. DECAPITATION.—I am part of a cart; behead me, I am part of a foot; behead me again, and I am a fish.

No. 12. SQUARE WORD.—1. Always. 2. A name. 3. A river. 4 To stagger.

### Results of First Week Puzzles.

No. 1. ENIGMA.—Blacksmith or farrier.

No. 2. GEOGRAPHICAL DIAMOND PUZZLE.

S
A P E
S P A I N
P I N
N

No. 3. BIOGRAPHICAL ACROSTIC.

R agla N
O thell O
D uma S
N ai L
E agl E
Y ar N

No. 4. BIRDS ENIGMATICALLY EXPESSED.—1. Kite. 2. Crane 3. Crow.

| Marks. | Total of Marks for July. | 1st Week of August. |
|---|---|---|
| A. C. K. | 15 | 4 |
| Alsie | 10 | 1 |
| Boadicea | 7 | 4 |
| Curly | 14 | 4 |
| Happy Eliza | 9 | — |
| Hercules | 11 | 2 |
| Jack | 13 | — |
| Geranium | 12 | — |
| Poodle | 8 | 2 |
| Thanet | 13 | 4 |
| Scrooge | 14 | 4 |
| Young Surgeon | 11 | — |
| Gem | 10 | 4 |
| W. R. M. | 3 | — |
| Judy | 7 | — |
| Ally | — | 4 |
| R. H | — | 4 |
| G. M. M. | — | 4 |
| Janette | — | 3 |
| Tynesider | — | 3 |

### Notice to Correspondents.

Owing to pressure on space the mark list was omitted last week, this week we insert total of marks for past month, with results of first week puzzles.

### Conditions.

I. Every paper sent in must be clearly written, and one side only must be used. All competitors must mark at the head of their papers the number of their competition.

II. Each paper must be signed by the author with his or her real name and address. A *nom de plume* may be added, if the writer does not desire to be referred to by us by his real name. In the case of all prizewinners, however, the real name and address will be published.

III. All communications relating to prize competitions should be addressed to "The Prize Editor, THE HOSPITAL, 38, Old Jewry, London, E.C." Competitors are requested not to include in letters, etc., to the Prize Editor communications on matters of other business. All letters must be fully prepaid.

IV. The Prize Editor of THE HOSPITAL is the sole judge of the competitions, and his decision is in all cases final and without appeal.

V. The Prize Editor reserves the right to publish any paper sent in, whether it receives a prize or not. He does not hold himself responsible for any MSS. sent, nor can he undertake to return rejected competitions.

VI.—All children resident at school are requested to forward their *home* address in addition to the school one, when entering for the "Children's Corner" Competitions.

**Notice to all Correspondents.**—N.B.—All letters referring to this page, which do not arrive at 38, Old Jewry, Cheapside, London, E.C., by the *first post on Thursdays*, and are not addressed PRIZE EDITOR, will in future be disqualified and disregarded.
No one will be permitted to win the Monthly Prizes twice in any one year, the Annual Prize being offered especially to prevent this.

